# Urinary miR-142-3p: a novel biomarker of the progression of IgA nephropathy

**DOI:** 10.3389/fimmu.2026.1715735

**Published:** 2026-05-11

**Authors:** Zhi-Yu Duan, Meng Zhang, Ru Bu, Qiu-Yue Zhang, Jie Wu, ShuWei Duan, ShuPeng Lin, Xi-Zhao Chen, WenJuan Wang, Xiang-Mei Chen, Zhe Feng, Guang-Yan Cai

**Affiliations:** Department of Nephrology, First Medical Center of Chinese People’s Liberation Army (PLA) General Hospital, National Key Laboratory of Kidney Diseases, National Clinical Research Center for Kidney Diseases, Beijing Key Laboratory of Kidney Diseases Research, Beijing, China

**Keywords:** IgA nephropathy, inositolpolyphosphate-5-phosphatase, kidney progression, mir-142-3p, urinary biomarker

## Abstract

**Background:**

The prognosis of IgA nephropathy (IgAN) varies greatly but tends to be poor. The purpose of the present study was to screen for urinary sediment miRNAs that could be used for the non-invasive prediction of IgAN progression and to explore the mechanisms explaining this.

**Methods:**

We studied two independent cohorts (2014–2015 and 2018–2022) to identify urinary sediment miRNAs that could be used to predict IgAN progression. Bioinformatic analysis and dual-luciferase experiments were used to identify target genes for miR-142-3p. The fibrotic phenotype of tubular epithelial cells was evaluated in HK-2 cells.

**Results:**

In both the training and validation cohorts, the urinary miR-142-3p expression in patients who showed IgAN progression was significantly higher than that in those who did not (*P* < 0.0001 and *P* = 0.003, respectively). Multivariate Cox regression analysis showed that high urinary miR-142-3p expression was an independent risk factor for IgAN progression (*P* < 0.001). Using the International IgA Nephropathy Prediction Tool (IIGANPT) as a reference, we replaced the pathologic indices in the IIGANPT model with the miR-142-3p expression and found that this did not reduce the predictive value of the model (*P* = 0.228). miR-142-3p is principally expressed in renal tubular epithelial cells, and the *in vitro* experiments showed that miR-142-3p influences PI3K–AKT pathway activity *via* inositol polyphosphate-5-phosphatase, thereby playing a role in this cell type’s fibrosis phenotype.

**Conclusions:**

Urinary miR-142-3p is a biomarker for the progression of IgAN and is involved in the exacerbation of renal fibrosis. Urinary miR-142-3p can be used to replace pathologic indices in the IIGANPT without reducing its predictive efficacy, implying that this modified tool could be used to non-invasively predict IgAN progression.

## Introduction

IgA nephropathy (IgAN) is one of the most common forms of primary glomerulonephritis worldwide (1). The prognosis of IgAN varies greatly but tends to be poor. A recent analysis of IgAN cohort data from the UK National Registry of Rare Kidney Diseases showed that most patients with IgAN are at risk of developing end-stage renal disease (ESRD) within their expected lifespan (2). Furthermore, IgAN significantly increases the risk of patient mortality and reduces their predicted lifespan (3). Predicting the progression of IgAN and intervening in a timely manner can help reduce the risk of ESRD developing in patients and reduce their mortality. Numerous researchers have attempted to establish predictive models for the progression of IgAN based on clinical and pathologic indices (4–6).

The International IgA Nephropathy Prediction Tool (IIGANPT) (6) is a currently recognized predictive model and its use is recommended in the 2021 Kidney Disease: Improving Global Outcomes (KDIGO) guidelines (7). IIGANPT is based on the Oxford Classification (MEST), incorporates clinical indices and treatment measures, and has a C-index of 0.82 for the full model, including race/ethnicity (6). However, the IIGANPT model has some limitations, including the requirement for pathologic indices, which precludes repeated assessment of disease progression during follow-up. IgAN is a chronic disease, but multiple sudden exacerbations and acute kidney injury (AKI) may occur during the course of the disease. Therefore, the renal pathology present at the onset of the disease cannot be used to accurately predict the progression of the disease at this time (8). Therefore, there is an urgent need to identify prognostic biomarkers of IgAN that could be used in clinical practice.

MiRNAs have been identified to be novel diagnostic and prognostic biomarkers for IgAN, owing to their high level of stability in renal tissue (9), the blood (10), and urine (11). Urinary miRNAs, which originate from renal cells, may represent alternative, non-invasive biomarkers of IgAN. Urinary miR-185-5p has been shown to have some predictive value for the progression of IgAN (12). However, multiple regression analysis did not show that it had independent predictive value. Furthermore, systematic studies investigating the relationship between urinary sediment miRNA expression and the progression of IgAN have been limited. Therefore, the purpose of the present study was to systematically screen for urinary sediment miRNAs that are associated with the progression of IgAN and determine whether they could be used to improve the IIGANPT.

## Materials and methods

### Study design and patients

We performed a two-stage study of patients from two independent cohorts. Urinary sediment samples were collected from patients who had received a diagnosis of primary IgAN on the basis of a renal biopsy performed at the Nephrology Department of the First Medical Center of the General Hospital of the People’s Liberation Army (PLA) of China between October 2013 and October 2014. A total of 179 patients were recruited to form a training cohort, of whom 163 completed at least 1 year of follow-up. Two patients with IgAN developed ESRD within the year following renal biopsy. In addition, 115 patients who received a diagnosis of non-IgAN glomerulonephritis on the basis of renal biopsy were recruited as a disease control group. Seventy patients in the disease control group were followed for more than 1 year, of whom 45 had membranous nephropathy (MN), 17 had minimal change nephropathy (MCN), 7 had focal segmental glomerulosclerosis (FSGS), and 1 had C3 glomerulopathy.

A total of 167 patients who underwent renal biopsy between October 2018 and April 2022 and were diagnosed with primary IgAN on this basis were recruited as a validation cohort. Of these, 146 completed at least 1 year of follow-up. In addition, 64 patients who had other types of glomerulonephritis during the same period of time were included as a disease control group, and 36 of these patients were followed for >1 year. These patients comprised 25 with MN, 4 with MCN, 5 with FSGS, and 2 with diabetic kidney disease (DKD).

The inclusion criteria were age ≥16 years, a diagnosis confirmed by renal biopsy, and an eGFR >15 mL/min/1.73 m^2^ before renal biopsy. The exclusion criteria were secondary IgAN (allergic purpura nephritis or lupus nephritis), concomitant other forms of glomerulonephritis (such as membranous nephropathy), and urinary tract infection or obstruction. There were no restrictions concerning the treatment of the patients. The study was approved by the Research Ethics Committee of the Chinese PLA General Hospital (Beijing, China) (approval numbers S2013-082–01 and S2018-206-01) and was performed in accordance with the principles of the Declaration of Helsinki. Informed consent was obtained from each of the patients before their inclusion in the study.

### Sample preparation and miRNA extraction

Each morning urine sample was centrifuged at 3,000 × g for 30 min and then 13,000 × g for 15 min at 4 °C. The urinary sediment obtained was stored at −80 °C until analyzed. TRIzol (Invitrogen, Waltham, MA, USA) was used to extract RNA from the urinary sediment, in accordance with the manufacturer’s protocol.

### Reanalysis of public miRNA microarray data

We reanalyzed the data obtained during the IgAN urinary sediment miRNA chip (GSE64306) analysis published in 2015 (13). Background correction, quantile normalization and probe-set summarization were performed with the Transcriptome Analysis Console (TAC) Software v4.01 (Thermo Fisher Scientific). Differentially expressed miRNAs were identified by one-way ANVA (built-in TAC algorithm) followed by Benjamin–Hochberg correction. A miRNA was considered significant when |fold change| > 1.5 and adjusted P < 0.05. No samples from this dataset were reused in the subsequent analyses of the present study.

### Reverse transcription–PCR analysis

Differential urinary sediment miRNA expression was validated by RT–PCR using a miRcute miRNA First-Strand cDNA Synthesis Kit (Tiangen Biotech, Beijing, China) and an miRcute miRNA qPCR Detection kit (Tiangen Biotech, Beijing, China). Urinary miRNA primers for hsa-U6 and hsa-miR-142-3p were purchased from Tiangen Biotech. RT-PCR was performed during the *in vitro* experiments using SuperScriptTM III First-Strand Synthesis SuperMix (ABI-Invitrogen, 11752050) and SYBRTM Select Master Mix (ABI-Invitrogen, 4472920). We used hsa-U6 (14) and glyceraldehyde-3-phosphate dehydrogenase (GAPDH) as reference genes. Relative miRNA expression was calculated using the 2^−ΔΔCt^ method.

### Clinical characteristics and renal histopathologic assessment of the patients

The clinical data for the patients were recorded at the time of renal biopsy, and included age, sex, mean arterial pressure (MAP), serum albumin concentration (the bromocresol green method), Scr (the enzymatic method), baseline eGFR, urine β-N-Acetyl-D-glucosaminidase (NAG) activity (colorimetric assay, Roche Diagnostics), 24-hour urinary protein quantification (the pyrogallol red molybdate method), and the presence or absence of hematuria. Hematuria was defined as >5 red blood cells/high-power field (assessed manually) or >28 red blood cells/µL (assessed using automated equipment). The Chronic Kidney Disease Epidemiology Collaboration (CKD–EPI) equation was used to calculate eGFR (15). The renal endpoint used to identify the progression of renal function was a decrease of ≥40% in eGFR and/or the development of ESRD or the need for kidney transplantation.

Two experienced pathologists graded the renal histopathology of the patients with IgAN using the Oxford classification system (MEST-C score) (16).

### *In situ* hybridization of miRNAs in renal tissue

Paraffin sections of renal tissue analyzed by light microscopy, see **Supplementary Methods** for details.

### Luciferase reporter assay

The luciferase activities were measured consecutively using a Dual-Luciferase Reporter Assay Kit (Beyotime Biotechnology, Naimen, China), see **Supplementary Methods** for details.

### Cell culture and transfection

HK-2 cells were obtained from Pricella (Wuhan, China) and cultured in Dulbecco’s-modified Eagle’s medium supplemented with 10% fetal bovine serum, 100 U/mL penicillin, and 0.1 mg/mL streptomycin. The cells were seeded into six-well plates and cultured until they reached 60%–70% confluence, then cultured in serum-free medium for an additional 12 h, before being transfected with 80 nM control mimic, miR-142-3p mimic, control inhibitor, or miR-142-3p inhibitor (GenePharma, Shanghai, China) using Lipofectamine 3000 (Invitrogen), in accordance with the manufacturer’s protocol.

HK-2 cells were transfected with siINPP5F (siRNA targeting inositol polyphosphate-5-phosphatase F)-1, siINPP5F-2, and siINPP5F-3 (Biomed, Wuhan, China) at a concentration of 80 nM using Lipofectamine 3000. The sequences of these siRNA were described in the **Supplementary Methods**.

HK-2 cells were cultured in serum-free conditions overnight, then incubated with 8 ng/mL recombinant human transforming growth factor β1 (TGF-β1) for 48 h to induce epithelial–mesenchymal transition. TGF-β1-stimulated HK-2 cells were then treated with 80 nM miR-142-3p inhibitor or siINPP5F-2 using Lipofectamine 3000 for an additional 48 h.

### Western blotting

HK-2 cells were homogenized in radioimmunoprecipitation assay lysis buffer (Thermo Fisher Scientific) and the protein concentration of each lysate was measured using a bicinchoninic acid assay (Thermo Fisher Scientific). The constituent proteins were separated by 10% sodium dodecyl sulfate–polyacrylamide gel electrophoresis (Thermo Fisher Scientific) and then transferred to polyvinylidene fluoride membranes (Millipore, Burlington, MA, USA). The membranes were blocked with 5% bovine serum albumin for 1 h, then incubated with the following primary antibodies overnight, see **Supplementary Methods** for details.

### Sample size calculation

Prior to the formal analysis, we performed an *a priori* power calculation using the public miRNA microarray dataset GSE64306 (13), which provides normalized signal intensities of urinary miR-142-3p from 6 progressors and 12 non-progressors. For this dataset, the inclusion criteria were: (i) patients with biopsy-confirmed primary IgA nephropathy; (ii) age between 20–50 years at renal biopsy; The exclusion criteria were: (i) treatment with steroids within 6 months prior to renal biopsy; (ii) fewer than eight glomeruli in light microscopy sections; (iii) concomitant chronic hepatic disease, diabetes, urinary/respiratory/gastrointestinal tract infection, rheumatoid arthritis, Henoch-Schönlein purpura, or systemic lupus erythematosus. The mean ± SD signal was 1.48 ± 0.66 vs 1.01 ± 0.16, yielding a Cohen’s d=1.19. For a two-sided α=0.05 and power=80%, a minimum of 12 subjects per group were required. Allowing for 15% loss to follow-up, we aimed to recruit ≥15 participants in each group. No sample from GSE64306 was reused in the current analyses.

### Statistical analysis

Statistical analysis was conducted using SPSS 24.0 (IBM, Inc., Armonk, NY, USA) or STATA 15.0 (Stata, USA) software. Graphics were prepared using Prism 5.01 software (GraphPad Corporation, USA). Normally distributed continuous data are summarized as mean ± standard deviation. Normality testing was conducted using the Shapiro-Wilk test or Kolmogorov-Smirnov test, depending on the sample size. The homogeneity of variance test, also known as the F-test, was used to determine whether the variance of a dataset was homogeneous. Comparisons between independent normally distributed continuous datasets with homogeneity of variance were conducted using the independent sample t-test or one-way ANOVA. Non-normally distributed data are summarized using the median and interquartile range and datasets were compared using the nonparametric Mann-Whitney U test. P<0.05 was considered to indicate statistical significance.

The 2^−ΔΔCt^ values for miR-142-3p expression were used to create a receiver operating characteristic (ROC) curve and calculate the AUC to evaluate the sensitivity and specificity of miR-142-3p for the prediction of IgAN progression. The Kaplan–Meier (K–M) curve was used to evaluate kidney disease-related survival. We used univariate and multivariate Cox proportional hazards models to evaluate the effect of urinary miR-142-3p expression level on the risk of IgAN progression. Using the IIGANPT as a reference model, we tested whether the inclusion of urinary miR-142-3p could improve the risk prediction achieved using this tool. In addition, by means of a comparison with the standard IIGANPT, we determined whether the pathologic indices in the IIGANPT could be replaced with urinary miR-142-3p expression without reducing its predictive performance. Multicollinearity among variables was assessed using variance inflation factors (VIF), with values <5.0 considered acceptable. No significant multicollinearity was detected in our models. The fit of the model was evaluated by calculating the Akaike information criterion (AIC) for the model with and without the inclusion of urinary miR-142-3p. The C-index, net reclassification improvement index (NRI), and integrated discrimination improvement index (IDI) were also calculated and used to estimate the increase in the predictive efficacy achieved using urinary miR-142-3p expression.

## Results

### Results of the high-throughput screening of urinary sediment miRNAs for associations with the progression of IgAN

We first reanalyzed the data obtained during the IgAN urinary sediment miRNA chip (GSE64306) analysis published in 2015 (13). The chip cohort consisted of 18 patients with primary IgAN, all of whom had completed follow-up. The median duration of follow-up for all of the patients in the cohort was 9.66 years (9.48–9.69 years). The cohort (**Supplementary Table 1**) was divided into a Progression group (n=6) and a Non-progression group (n=12), according to whether an endpoint event (a decrease of ≥40% in the eGFR and/or the development of ESRD or the need for kidney transplantation) occurred.

Analysis of the miRNA chip data revealed a total of 36 differentially expressed miRNAs between the Progression and Non-progression groups (**Supplementary Table 2**). In addition, we analyzed data from a 2021 next-generation sequencing study (GSE141344) which identified 36 miRNAs that were differentially expressed (*P* < 0.05) between an IgAN Progression group and a Non-progression group (17). We identified two miRNAs that demonstrated similar trends in the results of the two studies that could be used to predict the progression of IgAN: miR-142-3p and miR-142-5p, both of which showed significantly higher expression levels in the Progression groups than in the Non-progression groups (**Supplementary Table 3**).

The expression levels of miR-142-3p and miR-142-5p have been shown to be closely related (18). In addition, in both sets of data, the *P*-value for miR-142-3p was lower.

### Urinary miR−142−3p expression in the training cohort

The clinical data (**Supplementary Table 4**) showed that patients in the IgAN Progression group had higher MAP, more marked proteinuria, and higher serum creatinine (Scr), but lower serum albumin concentration and eGFR than those in the IgAN Non-progression group. With respect to the pathologic data, the proportions of patients with M1, S1, and T1-T2 grades were higher in the IgAN Progression group then in the IgAN Non-progression group, but there were no significant differences in the E and C grades between the groups. There was no significant difference in clinical data between the Progression and Non-progression disease control groups in the training cohort.

Urinary miR-142-3p was positively correlated with urinary protein quantification in IgAN patients (r=0.150, P = 0.046), while negatively correlated with urinary osmotic pressure (r=-0.252, P = 0.003) and serum albumin (r=-0.171, P = 0.023). However, there was no significant correlation between urinary miR-142-3p and eGFR, serum creatinine, M-grade, E-grade, S-grade, T-grade, and C-grade.

The expression of miR-142-3p in the Progression group was significantly higher than that in the Non-progression group [2.176 (1.169–4.182) vs. 0.698 (0.282–1.397), P<0.0001, respectively]. The AUC of the ROC curve was 0.796. The sensitivity for the detection of IgAN progression was 75.5% and the specificity was 70.5% (**Supplementary Table 5**). By contrast, miR-142-3p levels did not significantly differ between Progression and Non-progression groups in patients with other glomerulonephritides [0.021 (0.005-0.084) vs 0.030 (0.009-0.169), P = 0.321]].

According to the median expression level of miR-142-3p (2^-ΔΔCt^), patients with IgAN were allocated to a High expression group (n=83) and a Low expression group (n=82). During the follow-up period, 10 patients (12%) in the Low expression group experienced an endpoint event, while 43 patients (52%) in the High expression group experienced an endpoint event. K–M survival analysis showed that the renal prognosis of the High expression group was significantly worse than that of the Low expression group (log-rank test, *P* < 0.0001, **Supplementary Figure 1**).

Univariate and multivariate Cox regression analysis (**Table 1**) both revealed that urinary miR-142-3p expression [hazard ratio (HR) per 1 standard deviation higher 10.004, 95% confidence interval (CI) 3.361–29.774, *P* < 0.001] was an independent predictor of the progression of IgAN, whether it was treated as a continuous or a categorical variable (**Table 2**).

**Table 1:** Results of the Cox regression analysis of IgAN progression.

Variables	Univariate cox regression			Multivariate cox regression		
	HR	95%CI	P value	HR	95%CI	P value
miR-142-3p	14.732	5.845-37.132	<0.001	10.004	3.361-29.774	<0.001
proteinuria	1.310	1.216-1.411	<0.001	0.763	0.626-0.928	0.007
Serum albumin	0.865	0.827-0.904	<0.001	0.870	0.807-0.938	<0.001
Serum creatinine	1.021	1.017-1.025	0.001	1.017	1.006-1.027	<0.001
eGFR	0.955	0.943-0.967	<0.001			
M stage	3.222	1.743-5.956	<0.001			
E stage	2.531	1.184-5.411	0.017			
S stage	2.238	1.122-4.463	0.022			
T stage	6.040	2.842-12.838	<0.001			
ACEI/ARB usage rate	0.365	0.196-0.680	0.001			

HR, hazard ratio; CI, confidence interval; ACEI, angiotensin-converting enzyme inhibitor; ARB, angiotensin receptor blocker.

**Table 2:** Results of the multivariable Cox regression analysis of the use of urinary miR-142-3p for the prediction of the risk of IgAN progression in the training cohort.

Variables	Progression %	Unadjusted HR (95% CI)	P value	Model 1^a^	P value	Model 2^b^	P value
Urinary miR-142-3p	32.12	14.732 (5.845-37.132)	<0.0001	9.369 (3.293-26.656)	<0.0001	10.004 (3.361-29.774)	<0.0001
Subgroup							
Low group	12.05	1 (Reference)		1 (Reference)		1 (Reference)	
High group	52.44	4.857 (2.428-9.716)	<0.001	3.948 (1.886-8.266)	<0.001	3.613 (1.701-7.674)	<0.001

HR, hazard ratio; CI, confidence interval.

^a^ Model 1, adjusted for serum creatinine, serum albumin, eGFR, proteinuria, and the use of a renin-angiotensin system inhibitor before biopsy.

^b^ Model 2, adjusted for the covariates in Model 1 plus the Oxford MEST-C score.

To clarify whether urinary miR-142-3p expression could be used to increase the value of other predictors of IgAN progression, we added urinary miR-142-3p expression to the IIGANPT and compared the predictive efficacy of this combination with that of the IIGANPT alone. The addition of miR-142-3p improved the C-index of the IIGANPT (0.896 *vs*. 0.881), but the difference was not statistically significant, probably because of the small sample size (*P* = 0.050). Compared with the IIGANPT, the NRI of the IIGANPT+miR-142-3p model was −0.316 (−0.633, 0.0004), which did not significantly differ (*P* = 0.050), and the IDI was −0.028 (−0.062, 0.006), which also did not significantly differ (*P* = 0.107, **Table 3**). In addition, we replaced the pathologic indices in the IIGANPT with urinary miR-142-3p expression and compared the predictive values of the two models. The C-index of the standard IIGANPT was 0.881 and that of the model comprising clinical indices+history of therapy+miR-142-3p expression (created by replacing the pathologic indices in the IIGANPT with urinary miR-142-3p expression) was similar (0.883, *P* = 0.228, **Table 3**).

**Table 3:** Comparison of the predictive efficacy of the IgAN progression prediction models.

Models	AIC	C-index (95% CI)	NRI (95% CI)	P value	IDI (95% CI)	P value
IIGANPT^a^	378.370	0.881 (0.837,0.925)	Reference		Reference	
IIGANPT+miR-142-3p^b^	367.037	0.896 (0.854,0.937)	-0.316 (-0.633,0.0004)	0.050	-0.028 (-0.062,0.006)	0.107
Clinical indicators+miR-142-3p+therapeutic indicators^c^	368.592	0.883 (0.841,0.924)	-0.176 (-0.499,0.148)	0.228	-0.017 (-0.09,0.055)	0.637

CI, confidence interval; AIC, Akaike information criterion; C-index, concordance index; NRI, net reclassification improvement index; IDI, integrated discrimination improvement index.

^a^ IIGANPT, which comprised age, proteinuria, MAP, eGFR, M-grade, E-grade, S-grade, T-grade, C-grade, the use of a renin–angiotensin system inhibitor before biopsy, the use of an immunosuppressive drug before biopsy, and race/ethnicity.

^b^ IIGANPT+miR-142-3p model.

^c^ Clinical indices+history of drug administration+miR-142-3p model, which comprised age, proteinuria, MAP, eGFR, the use of a renin–angiotensin system inhibitor before biopsy, the use of an immunosuppressive drug before biopsy, and urinary miR-142-3p expression.

There were no significant differences in the values of general parameters between the Progression and Non-progression disease control groups in the training cohort (**Supplementary Table 6**). In addition, there was no significant difference in the urinary miR-142-3p expression of the Progression and Non-progression disease control groups in the training cohort [0.135 (0.030–0.549) *vs*. 0.195 (0.058–1.101), respectively, *P* = 0.321].

### Urinary miR−142−3p expression in the validation cohort

In the validation cohort, the patients in the Progression group had greater proteinuria and higher Scr, but less hematuria and lower eGFR than those in the Non-progression group. With respect to the pathologic data, patients in the Progression group were more likely to have T1–T2 grades, but there were no significant differences in the proportions of M, E, S, and C grades between the two groups (**Supplementary Table 7**). The miR-142-3p expression of the Progression group was significantly higher than that of the Non-progression group [4.228 (0.428–10.543) *vs*. 0.321 (0.095–0.960), *P* = 0.003]. The AUC of the ROC curve was 0.779. The sensitivity for the detection of IgAN progression was 50.0% and the specificity was 97.1% (**Supplementary Table 8**).

The patients in the Progression disease control group of the validation cohort were older than those in the Non-progression disease control group, but there were no significant differences in the other indices (**Supplementary Table 9**). In the validation cohort, there was no significant difference in the urinary miR-142-3p expression levels of the Progression and Non-progression disease control groups [0.1074 (0.0550–6.3387) *vs*. 0.1725 (0.0375–0.9770), respectively; *P* = 0.732)].

### Results of the *in-situ* hybridization analysis of miR-142-3p in renal tissue

**Figure 1:**
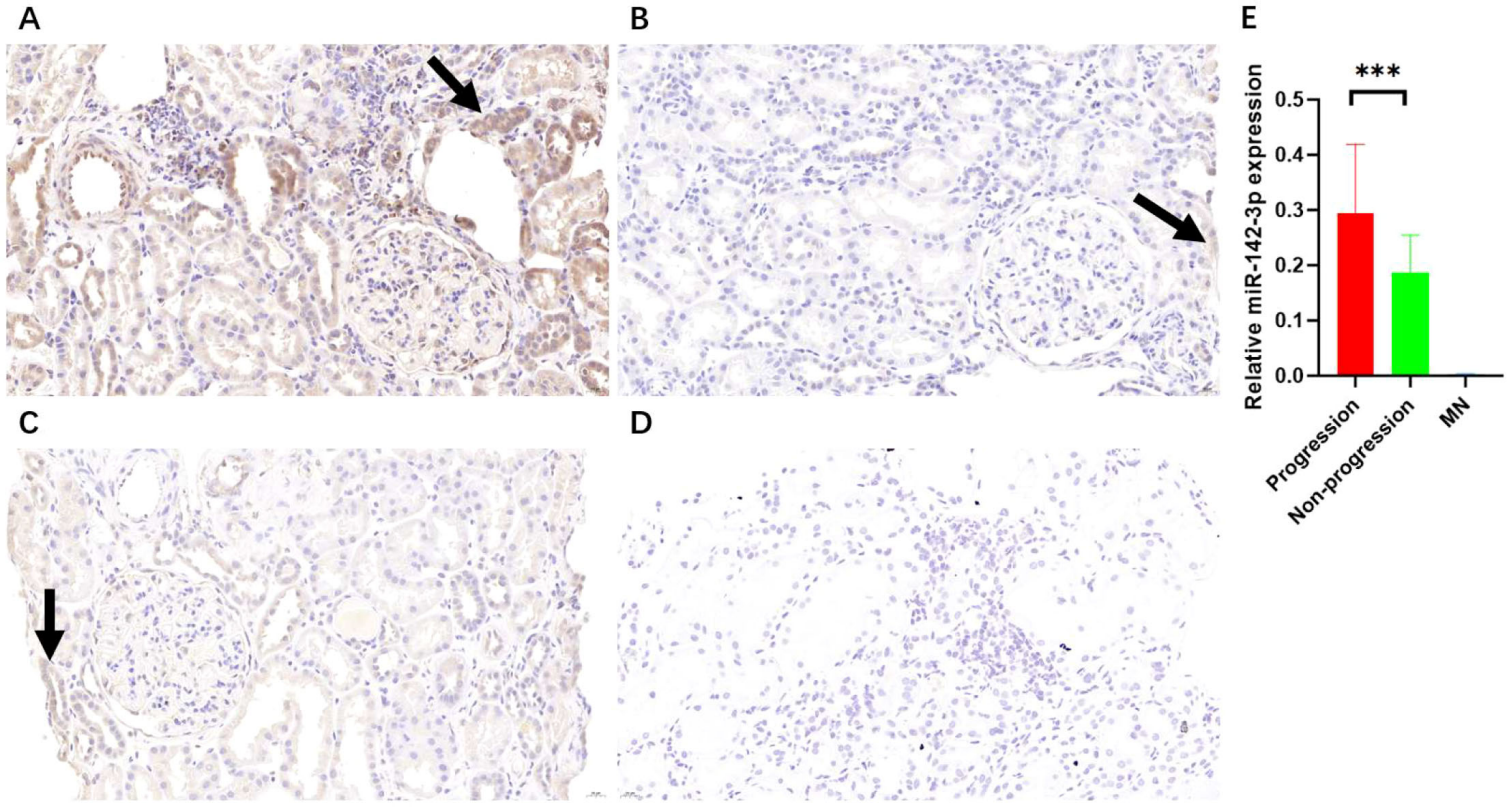
Images of the results of *in situ* hybridization for miR-142-3p in renal tissue. **(A)** IgAN Progression group (n=3); **(B)** Non-progression group (n=3); **(C)** MN group (n=3); **(D)** Negative control group (n=3); **(E)** Quantitative analysis of staining intensity using ImageJ (mean ± SD: Progression group 0.295 ± 0.124 vs. Non-progression group 0.187 ± 0.068 vs. MN group 0.0025 ± 0.0019, P<0.001). Original magnification, ×400, scale bar=20 µm. ***, *P* < 0.001. Arrows indicate miR-142-3p-positive cells (renal tubular epithelial cells indicated by arrows).

The relative expression level of miR-142-3p in the IgAN Progression group was significantly higher than that in the Non-progression group (0.295 ± 0.124 vs 0.187 ± 0.068, *P* = 0.007). The relative expression level of miR-142-3p in the negative control group was significantly lower than that in the Progression group and the Non-progression group (0.0025 ± 0.0019, *P* < 0.001). Whether samples were obtained from patients in the IgAN Progression group, the Non-progression group, or the MN group, miR-142-3p staining was mainly in the renal tubules, and the staining intensity in the glomeruli was significantly lower (**Figure 1**).

### INPP5F is a target gene of miR-142-3p

Using DIANA-microT target gene prediction software, we identified inositol polyphosphate-5-phosphatase F (INPP5F) as a potential target gene of miR-142-3p. The mass spectrometry data for the kidneys of patients with IgAN were reanalyzed (https://www.iprox.cn, PXD032710) (19). In this dataset, there were 6 patients in the Progression group and 48 in the Non-progression group, and there were no significant differences in the demographic, clinical, or pathologic data for the two groups (**Supplementary Table 10**). However, the protein expression of INPP5F in the Progression group was significantly lower than that in the Non-progression group [0.525 (0.444–0.545) *vs*. 0.939 (0.721–1.533), *P* = 0.007]. In addition, according to the Human Protein Atlas database (https://www.proteinatlas.org), INPP5F is mainly expressed in renal tubular epithelial cells in humans, as for miR-142-3p.

**Figure 2:**
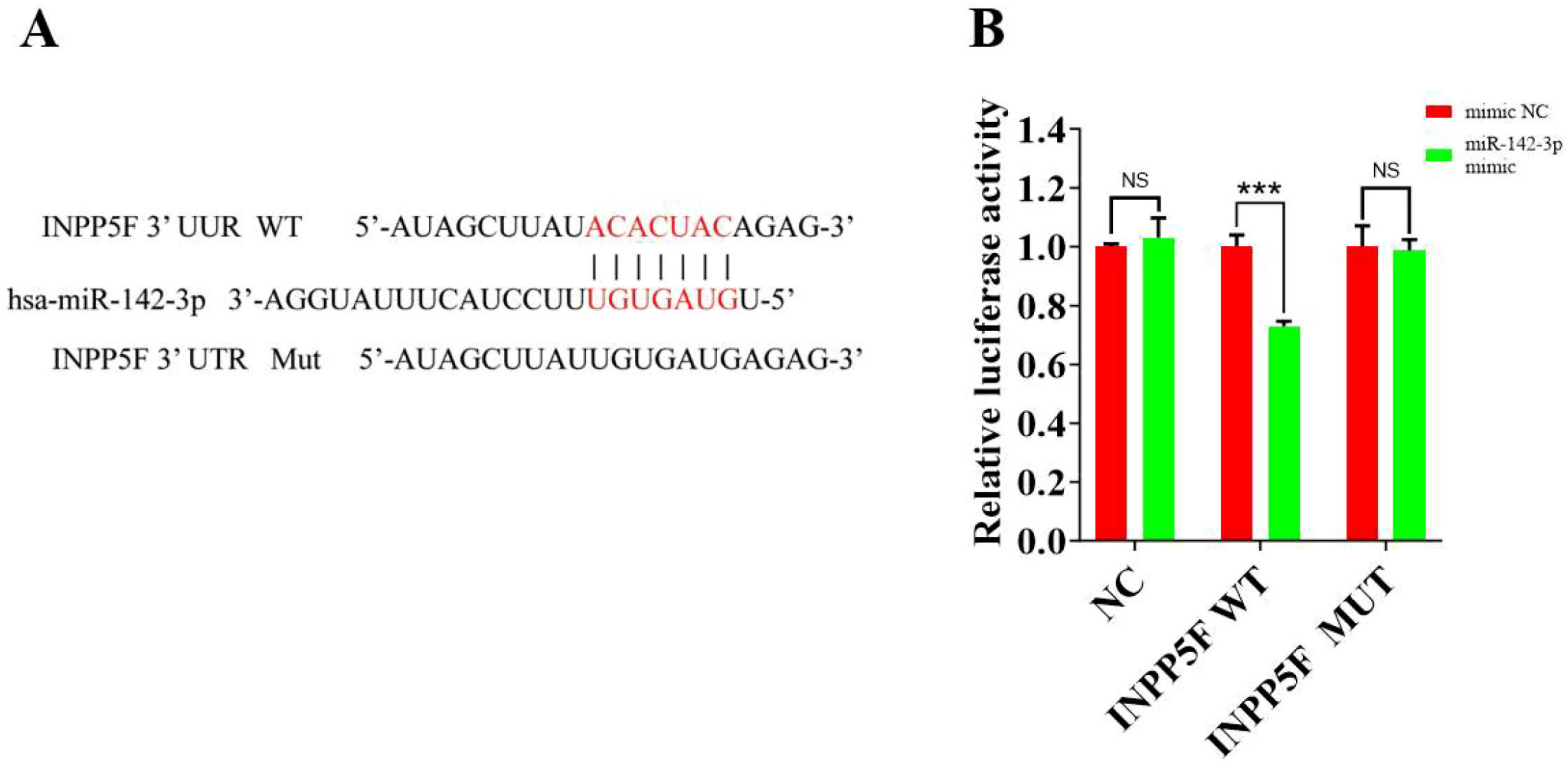
Effect of miR-142-3p on the expression of INPP5F (dual-luciferase reporter assay). **(A)** Prediction of the miR-142-3p binding site in INPP5F mRNA 3′- UTR. **(B)** Results of the dual luciferase assay of the effect of miR-142-3p on INPP5F expression. Experiments were performed in HK-2 cells; NS, no significant difference; ***P < 0.001. NC, negative control (n=3), WT, wild-type (n=3); MUT, mutant (n=3).

**Figure 2A** shows the miR-142-3p binding site in the 3′-UTR of the INPP5F mRNA, as predicted by the DIANA-microT target gene database. We found that the miR-142-3p mimic significantly reduced INPP5F expression in wild-type-transfected cells, but there were no significant differences in its expression in the other groups of cells (**Figure 2**).

### miR-142-3p promotes fibrosis and activates the PI3K–AKT pathway in HK-2 cells

We next transfected HK-2 cells with the control mimic, the miR-142-3p mimic, or no construct and then measured the expression of miR-142-3p. The expression in the miR-142-3p mimic-transfected cells was significantly higher than that in the other two groups, indicating successful transfection (*P* < 0.001). Furthermore, the mRNA expression of INPP5F in the miR-142-3p-transfected cells was significantly lower than that in the other two groups of cells (*P* = 0.0013, **Supplementary Figure 2**).

**Figure 3:**
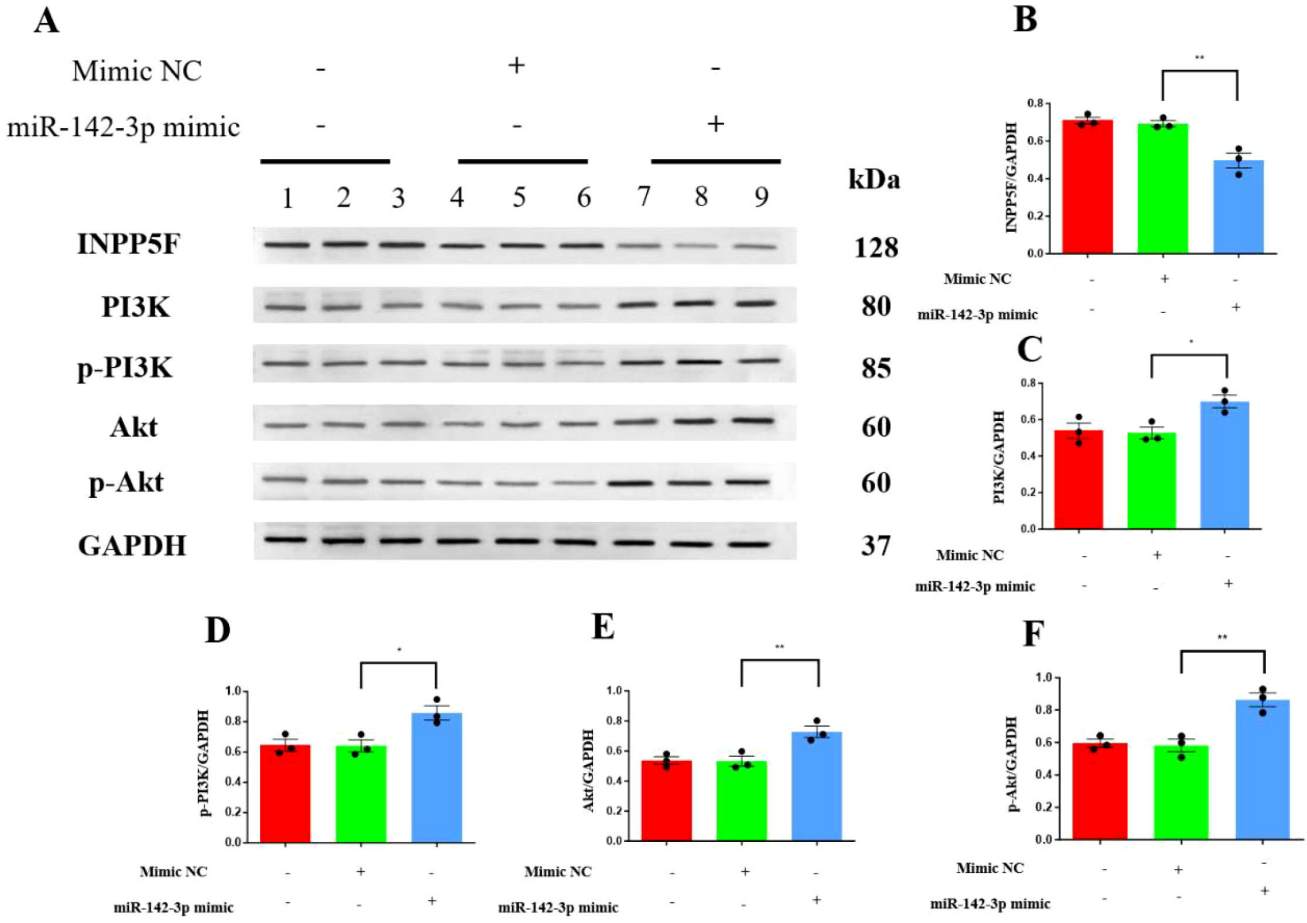
Transfection with miR-142-3p mimic resulted in upregulation of PI3K, p-PI3K, AKT and p-AKT in HK-2 cells. **(A)** Western blots of INPP5F, PI3K, p-PI3K, AKT, and p-AKT in HK-2 cells; **(B)** Western blotting data for INPP5F; **(C)** Western blotting data for PI3K; **(D)** Western blotting data for p-PI3K; **(E)** Western blotting data for AKT; and **(F)** Western blotting data for p-AKT. Quantitative data represent densitometric analysis normalized to GAPDH. *P < 0.05, **P < 0.01.

The miR-142-3p mimic-transfected cells also showed significantly lower expression of the target protein INPP5F (P = 0.0024), whereas the expression levels of phosphatidylinositol 3-kinase (PI3K) (*P* = 0.0264), phosphorylated PI3K (p-PI3K, *P* = 0.0151), protein kinase B (AKT) (*P* = 0.0081), and phosphorylated Akt (p-Akt, *P* = 0.0024) were all significantly higher (**Figure 3**).

**Figure 4:**
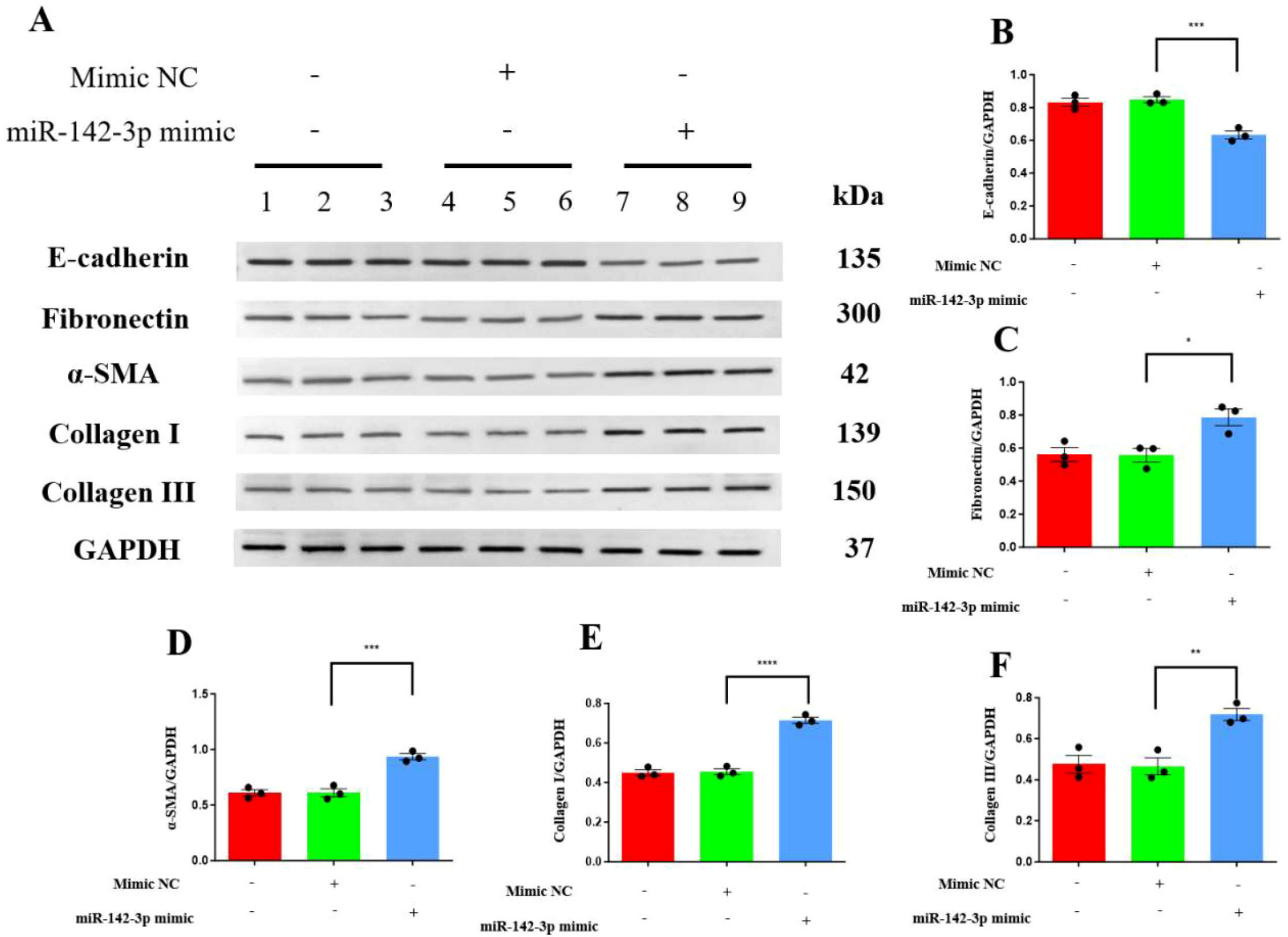
Overexpression of miR-142-3p induced epithelial-mesenchymal transition in HK-2 cells, characterized by decreased E-cadherin and increased fibronectin, α-SMA, collagen I, and collagen III. **(A)** Western blots of E-cadherin, fibronectin, α-SMA, collagen I, and collagen III; **(B)** Western blotting data for INPP5F; **(C)** Western blotting data for fibronectin; **(D)** Western blotting data for α-SMA; **(E)** Western blotting data for collagen I; and **(F)** Western blotting data for collagen III. Quantitative data represent densitometric analysis normalized to GAPDH. *P < 0.05, **P < 0.01, ***P < 0.001, ****P < 0.0001.

In addition, the overexpression of miR-142-3p caused a significant reduction in the expression of E-cadherin in the miR-142-3p mimic-transfected cells (*P* < 0.001) but caused significant increases in the expression of indicators of a profibrotic phenotype [fibronectin (*P* = 0.017), α-SMA (*P* < 0.001), collagen I (*P* < 0.0001), and collagen III (*P* = 0.0056)] (**Figure 4**).

After transfecting HK-2 cells with miR-142-3p mimic, control mimic, miR-142-3p inhibitor, or control inhibitor, PCR analysis (**Supplementary Figure 3**) showed that the expression of miR-142-3p was significantly higher in the miR-142-3p mimic-transfected cells (*P* < 0.001) and significantly lower in the miR-142-3p inhibitor-transfected cells (*P* < 0.001), indicating successful transfection of each.

In addition, we transfected three siRNAs (INPP5F-1, -2, and -3) and a control siRNA into groups of HK-2 cells. siRNA-INPP5F-2 was used in subsequent experiments by PCR analysis and western blotting (**Supplementary Figure 4**).

HK-2 cells were transfected with siRNA-INPP5F-2, treated with TGF-β1, treated with TGF-β1 and transfected with miR-142-3p inhibitor, or treated with TGF-β1 and transfected with miR-142-3p inhibitor and siRNA-INPP5F-2. Subsequent PCR analysis showed that the expression of miR-142-3p in TGF-β1-treated cells was higher than that in control cells (*P* = 0.0050), but that additional transfection with the miR-142-3p inhibitor significantly reduced miR-142-3p expression (*P* = 0.0103, **Supplementary Figure 5**). In addition, compared with the control group, both the siRNA-INPP5F-2-transfected cells (*P* < 0.001) and the TGF-β1-treated cells (*P* < 0.001) showed significantly lower expression of INPP5F mRNA. Additional transfection with miR-142-3p inhibitor significantly increased the expression of INPP5F mRNA (*P* = 0.0058, **Supplementary Figure 5**).

**Figure 5:**
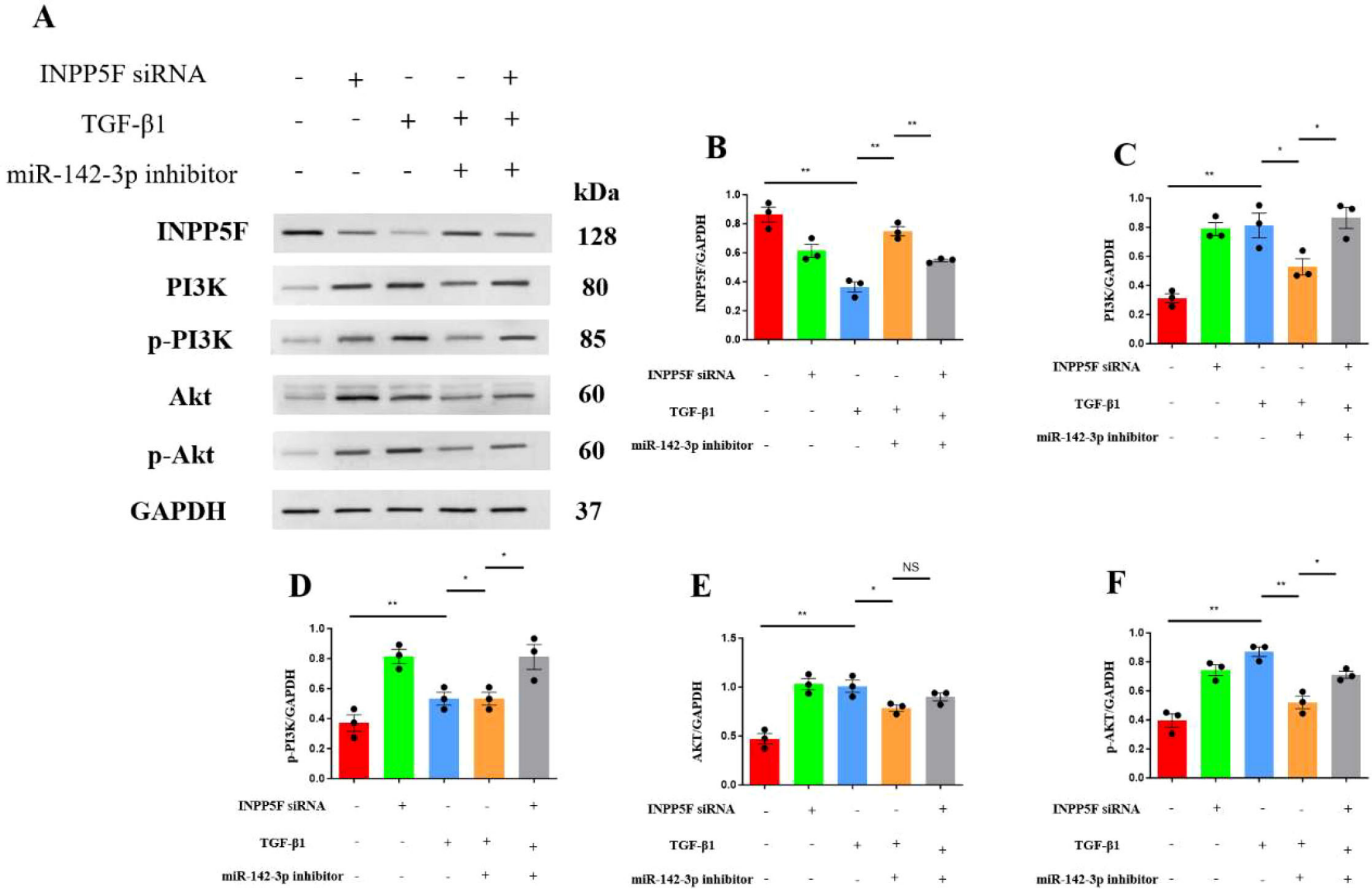
Inhibition of miR-142-3p attenuated TGF-β1-induced PI3K-AKT activation in HK-2 cells. **(A)** Western blots of INPP5F, PI3K, p-PI3K, AKT, and p-AKT; **(B)** Western blotting data for INPP5F; **(C)** Western blotting data for PI3K; **(D)** Western blotting data for p-PI3K; **(E)** Western blotting data for AKT; and **(F)** Western blotting data for p-AKT. Quantitative data represent densitometric analysis normalized to GAPDH. *P < 0.05; **P < 0.01; NS, no significant difference.

Western blotting showed that TGF-β1 treatment significantly reduced the expression of INPP5F (*P* = 0.0014), but concurrent miR-142-3p inhibitor transfection significantly increased the expression of INPP5F (*P* = 0.0013). siRNA-INPP5F-2 transfection prevented the increase in INPP5F expression induced by miR-142-3p inhibitor (*P* = 0.0038). In addition, TGF-β1 treatment significantly increased both the expression of PI3K (*P* = 0.0053) and AKT (*P* = 0.0029) and their phosphorylation (*P* = 0.0049 and *P* = 0.0011, respectively). miR-142-3p inhibitor transfection reduced the expression of PI3K (*P* = 0.0495) and AKT (*P* = 0.0335) and their phosphorylation (*P* = 0.0162 and *P* = 0.0030, respectively) in TGF-β1-treated cells. Finally, siRNA-INPP5F-2 transfection prevented the miR-142-3p inhibitor-induced reduction in PI3K (*P* = 0.0211) expression and the reductions in PI3K (*P* = 0.0403) and AKT (*P* = 0.0182) phosphorylation, and there was a similar trend for AKT expression (*P* = 0.0903) (**Figure 5**).

**Figure 6:**
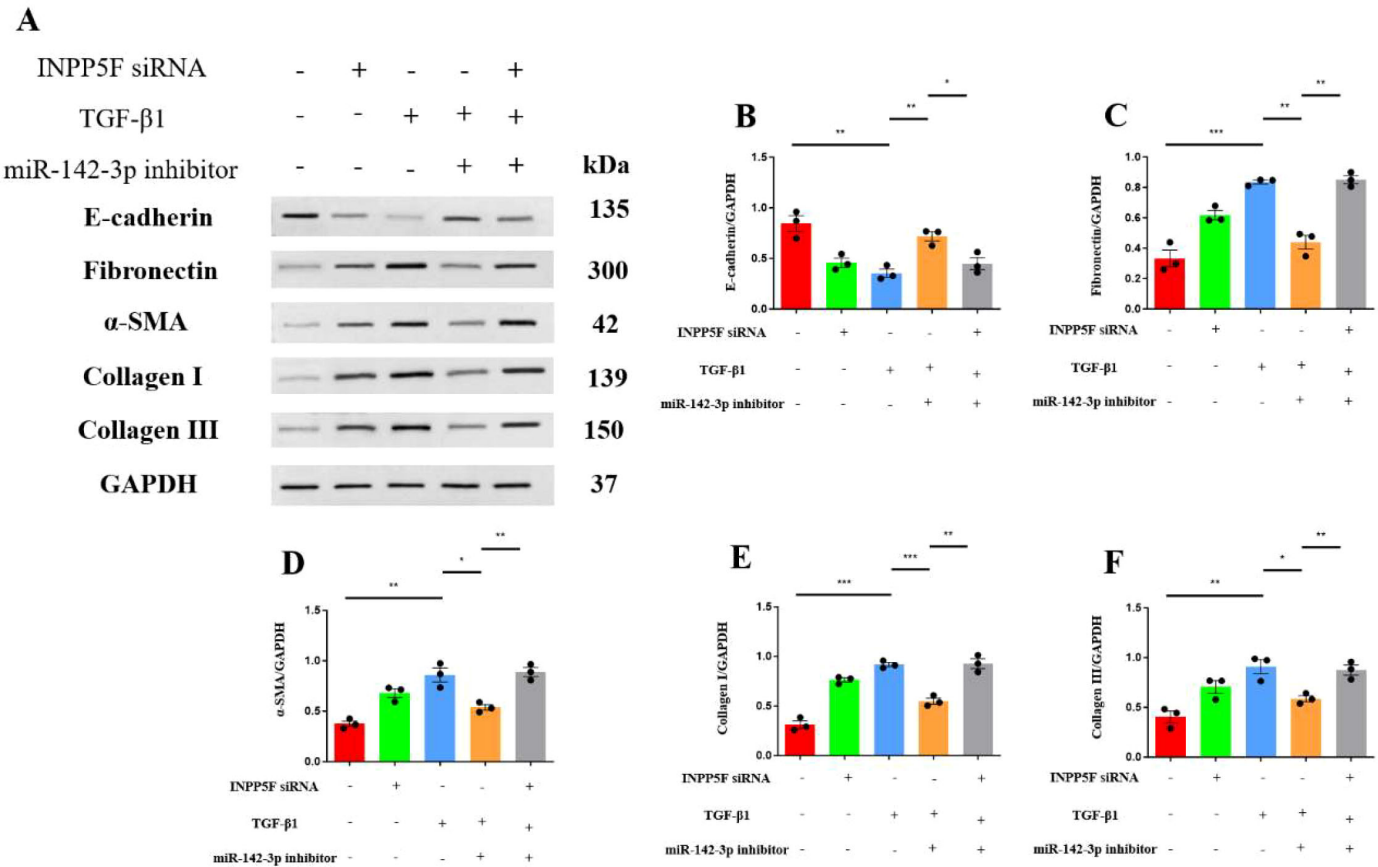
Knockdown of INPP5F reversed the protective effects of miR-142-3p inhibition against TGF-β1-induced fibrosis in HK-2 cells. **(A)** Western blots of E-cadherin, fibronectin, α-SMA, collagen I, and collagen III expression in HK-2 cells; **(B)** Western blotting data for E-cadherin; **(C)** Western blotting data for fibronectin; **(D)** Western blotting data for α-SMA; **(E)** Western blotting data for collagen I; and **(F)** Western blotting data for collagen III. Quantitative data represent densitometric analysis normalized to GAPDH. *P < 0.05, **P < 0.01, ***P < 0.001.

In addition, TGF-β1 treatment significantly reduced the expression of E-cadherin (*P* = 0.0048) (**Figure 6**). However, miR-142-3p inhibitor transfection significantly increased the expression of E-cadherin in TGF-β1-treated cells (*P* = 0.0040). Furthermore, siRNA-INPP5F-2 prevented the miR-142-3p inhibitor-induced increase in E-cadherin expression (*P* = 0.0231). In addition, TGF-β1 treatment significantly increased the expression of proteins associated with fibrosis [fibronectin (*P* < 0.001), α-SMA (*P* = 0.0030), collagen I (*P* < 0.001), and collagen III (*P* = 0.0058)]. miR-142-3p inhibitor transfection significantly reduced the expression of fibronectin (*P* = 0.0012), α-SMA (*P* = 0.0127), collagen I (*P* < 0.001), and collagen III (*P* = 0.0150) in TGF-β1-treated cells. Furthermore, siRNA-INPP5F-2 prevented the reductions in fibronectin (*P* = 0.0016), α-SMA (*P* = 0.0028), collagen I (*P* = 0.0028), and collagen III (*P* = 0.0082) expression induced by the miR-142-3p inhibitor (**Figure 6**).

## Discussion

In the present study, we identified 36 urinary sediment miRNAs that could be used to predict the progression of IgAN. Two potential biomarkers, including miR-142-3p, were identified by means of an analysis of these data alongside those generated by the next-generation sequencing of another set of miRNA data relating to IgAN progression (17). Analysis of the training cohort and validation cohort showed that the expression of miR-142-3p in urinary sediment is an independent risk factor for the progression of IgAN. Although the addition of urinary sediment miR-142-3p expression to the IIGANPT (6) did not significantly improve its predictive value, because of the small sample size and other factors, we have provided evidence that miR-142-3p could replace the pathologic indices in the IIGANPT without reducing its predictive efficacy, thereby providing a non-invasive method of predicting IgAN progression. Finally, we have also shown that urinary sediment miR-142-3p is not a predictor of the progression of glomerulonephritis in patients other than those with IgAN, indicating that urinary sediment miR-142-3p may have some specificity in its use for the prediction of kidney disease progression. This disease-specificity distinguishes miR-142-3p from generic markers of kidney injury such as proteinuria or eGFR decline, which are common across various renal diseases.

Many researchers have attempted to identify miRNA biomarkers of IgAN progression. To date, the reported miRNA biomarkers of IgAN progression have principally been identified in extracellular vesicles and renal tissue (17, 20–22), and there have been few studies of urinary miRNAs. In 2023, Korean researchers discovered that urinary exosomal miR-16-5p, miR-199a-3p, and miR-335-3p are biomarkers of IgAN progression, with miR-199a-3p having the highest AUC value of 0.749 (20). However, the method of separating urinary exosomes is more complex than that used to obtain urinary sediment, making the former impractical for large-scale clinical use. In 2021, Kidney International published an article by researchers in the UK who identified four miRNA biomarkers (miR-150-5p, miR-155-5p, miR-146b-5p, and miR-135a-5p) that could be used to predict the progression of IgAN, and miR-150-5p was found to have the highest predictive value, with an AUC value of 0.8 (17). However, because it is expressed in kidney tissue, it is not suitable for use as a non-invasive predictor, given that a large volume of kidney tissue is required to quantify miRNAs, potentially necessitating additional tissue biopsies. In 2023, Indian researchers discovered that the plasma miR-21 level could also be used to predict the progression of IgAN, but the mean duration of follow-up in this study was only 21.5 months and the endpoint event used was a doubling of Scr, which can be a relatively short-term change (23). In the present study, we have confirmed that urinary sediment miR-142-3p is a biomarker for the progression of IgAN using three independent cohorts: a miRNA chip cohort, a training cohort, and a validation cohort. Furthermore, the median duration of follow-up for the miRNA chip cohort was 9.66 years and that of the training cohort was 5.93 years, which implies that urinary sediment miR-142-3p is a useful predictor of the long-term progression of IgAN.

The IIGANPT is a current risk prediction model for the progression of IgAN (17) and its use is recommended in the 2021 KDIGO guidelines (7). This model integrates pathologic, clinical, and treatment-related indices, and the pathologic indices are important components of this. The need for pathologic indices means that it is not possible to repeatedly evaluate the changes in the patient’s condition over the long term. Therefore, the identification of non-invasive biomarkers that could be used in place of these pathologic indices is a key issue for renal practice (8). A study of 762 patients with IgAN that had a median duration of follow-up of 65 months showed that urinary IL-6 concentration is an independent risk factor for the progression of IgAN. In addition, the C-index for the use of urinary IL-6 concentration in combination with clinical indices was as high as 0.84 (24). However, the IIGANPT was not used as a reference model in this study, and therefore further research is needed to determine whether urinary IL-6 concentration could be used in place of the pathologic indices in the IIGANPT. However, the study has shown that the C-indices for the clinical indices+treatment history+miR-142-3p model and the IIGANPT are similar, suggesting that urinary miR-142-3p concentration could be used in place of the pathologic indices in the IIGANPT.

The miR-142 hairpin can generate the “guide chain” miR-142-3p and the sister “passenger chain” miR-142-5p. The expression levels of miR-142-3p and miR-142-5p are closely related, but they have different target genes (18). miR-142-3p has been reported to be a biomarker for the development and prognosis of many diseases, such as psoriasis (25), acute lymphoblastic leukemia (26), and unstable angina (27). In a study of renal cancer, extracellular vesicles derived from renal cancer stem cells were shown to induce endoplasmic reticulum stress and apoptosis in renal tubular epithelial cells through miR-142-3p, ultimately leading to a decline in renal function (28). Another study showed that the expression of miR-142-3p in the kidneys of an IgAN Progression group is significantly higher than that of a Non-progression group (17). In addition, the expression of miR-142-3p in the renal tissue of a CKD Progression group was found to be significantly higher than that of a Non-progression group (22). A previous cohort study and meta-analysis showed that renal miR-142-3p expression is a biomarker of the severity of renal tubular atrophy/interstitial fibrosis (29, 30), which is the most reliable histologic biomarker of poor prognosis in patients with IgAN (31). Therefore, we speculate that the ability to predict the progression of IgAN using urinary sediment miR-142-3p may be explained by its role in the progression of renal fibrosis.

INPP5F was identified as a target gene of miR-142-3p, and miR-142-3p was found to be principally expressed in renal tubular epithelial cells. IgAN renal mass spectrometry data also confirmed that the expression of INPP5F in patients that show progression of disease is significantly lower than that in those who do not (19). INPP5F is an SAC phosphatase, inhibiting the conversion of phosphatidylinositol diphosphate (PIP2) to phosphatidylinositol triphosphate (PIP3) and promoting the conversion of PIP2 to phosphatidylinositol phosphate (PIP), thereby inhibiting the PI3K–AKT signaling pathway, by virtue of its SAC phosphatase domain (32). A previous study of stress-induced hypertension also showed that INPP5F inhibits the PI3K–AKT pathway, because overexpression of INPP5F reduces the blood pressure, sympathetic nervous system excitability, and neuronal excitability of patients with stress-induced hypertension by inhibiting the PI3K–AKT pathway (33). Our data position the miR-142-3p/INPP5F/PI3K-AKT axis as a critical downstream mediator of TGF-β-induced fibrosis. We demonstrate that TGF-β1 concurrently upregulates miR-142-3p, which in turn suppresses INPP5F to amplify PI3K-AKT activation. This creates a positive feedback loop whereby TGF-β1-induced upregulation of miR-142-3p results in INPP5F suppression, PI3K-AKT pathway activation, and amplification of the fibrotic response. The clinical relevance of INPP5F dysfunction is underscored by Lowe syndrome, caused by OCRL1/INPP5F mutations (34), where patients develop progressive renal fibrosis due to defective phosphoinositide metabolism. This human genetic disorder validates our mechanistic finding that INPP5F deficiency promotes renal fibrosis via PI3K-AKT dysregulation. In the present study, we found that increasing the expression of miR-142-3p in HK-2 cells activates the PI3K–AKT pathway and promotes the generation of a profibrotic phenotype. Furthermore, reducing the expression of miR-142-3p increases the expression of INPP5F, inhibits the PI3K–AKT pathway, and prevents the profibrotic phenotype; and inhibition of the expression of INPP5F can reverse this. These results suggest that miR-142-3p plays a role in the progression of IgAN by inhibiting the expression of its target gene INPP5F, activating the PI3K–AKT pathway, and inducing a profibrotic phenotype.

The central role of PI3K-AKT signaling in tissue fibrosis extends beyond the kidney to other organ systems. Recent studies have demonstrated that biomimetic scaffolds promote stem cell differentiation and cardiac repair by modulating the PI3K/AKT pathway (35), underscoring the conserved function of this signaling axis in tissue remodeling. Similarly, the MrgD receptor has been shown to regulate smooth muscle cell proliferation and collagen deposition via AKT signaling in vascular disease (36). These findings parallel our observation that miR-142-3p-mediated INPP5F suppression activates PI3K-AKT to promote renal tubular epithelial cell fibrosis. Together, these studies support the miR-142-3p/INPP5F/PI3K-AKT axis as a potential therapeutic target not only for IgAN but potentially for fibrotic diseases across multiple organ systems.

The clinical significance of urinary miR-142-3p extends beyond its statistical independence from established risk factors. The modest correlation with proteinuria and serum albumin, combined with the lack of correlation with eGFR and Oxford MEST-C scores, positions miR-142-3p as a mechanistically distinct biomarker that captures active disease processes rather than accumulated structural damage or functional impairment. This distinction is clinically meaningful for two primary reasons. First, unlike eGFR—which merely reflects current kidney function—miR-142-3p appears to reflect ongoing pathophysiological activity in the renal tubulointerstitium, as evidenced by its principal expression in tubular epithelial cells and its role in promoting fibrosis via the INPP5F/PI3K-AKT pathway. Second, unlike MEST-C scores—which represent static, biopsy-derived structural lesions—urinary miR-142-3p can be longitudinally monitored to track disease dynamics without repeated invasive procedures. Thus, miR-142-3p complements, rather than replaces, existing prognostic tools, offering a non-invasive window into active tubulointerstitial injury inaccessible through routine clinical or pathological assessment.

There were some limitations to the present study. Firstly, owing to the retrospective nature of the study, some indices, such as the urinary protein-to-creatinine ratio and the serum cystatin C concentration, could not be obtained. Secondly, it was a single-center retrospective study, and therefore the value of using urinary miR-142-3p expression in the prediction of IgAN progression requires further validation at other centers and in cohorts of other ethnicities.

## Conclusions

Urinary sediment miR-142-3p is a biomarker for the progression of IgAN and may be used in place of the pathologic indices in the IIGANPT without reducing its predictive efficacy, rendering it a non-invasive means of predicting IgAN progression. miR-142-3p activates the PI3K–AKT pathway and the development of a profibrotic phenotype in renal epithelial cells through its target gene INPP5F, thereby playing a role in the progression of IgAN.

## Data Availability

The datasets presented in this study can be found in online repositories. The names of the repository/repositories and accession number(s) can be found below: https://www.ncbi.nlm.nih.gov/geo/query/acc.cgi?acc=GSE64306 (GSE64306), https://www.ncbi.nlm.nih.gov/geo/query/acc.cgi?acc=GSE141344 (GSE141344), and https://www.iprox.cn (PXD032710). The raw clinical data supporting the conclusions of this article are not publicly available due to patient privacy and ethical restrictions but can be made available upon reasonable request to the corresponding author.

## Glossary

IgAN: IgA nephropathy
ESRD: End-stage renal disease
IIGANPT: International IgA Nephropathy Prediction Tool
KDIGO: Kidney Disease: Improving Global Outcomes
AKI: Acute kidney injury
AUC: Area under curve
eGFR: Estimated glomerular filtration rate
MN: Membranous nephropathy
MCN: Minimal change nephropathy
FSGS: Focal segmental glomerulosclerosis
DKD: Diabetic kidney disease
LC-MS/MS: Chromatography-tandem mass spectrometry
RT-PCR: Reverse transcription polymerase chain reaction
MAP: Mean arterial pressure
GAPDH: Glyceraldehyde-3-phosphate dehydrogenase
NAG: β-N-Acetyl-D-glucosaminidase
INPP5F: Inositol polyphosphate-5-phosphatase F
siRNA: Small interfering RNAs
TGF-β1: Transforming growth factor beta1
α-SMA: Alpha-smooth muscle actin
ROC: Receiver operating characteristic
K-M: Kaplan-Meier
AIC: Akaike information criterion
C-index: Concordance index
NRI: Net reclassification improvement index
IDI: Integrated discrimination improvement index
HR: Hazard ratios
CI: Confidence interval
ACEI: Angiotensin-converting enzyme inhibitor
ARB: Angiotensin receptor blocker
NS: No statistical difference
NC: Negative control
PIP2: Phosphatidylinositol diphosphate
PIP3: Phosphatidylinositol triphosphate
PIP: Phosphatidylinositol phosphate.
