# Supplementary material for: Urinary miR-142-3p: a novel biomarker of the progression of IgA nephropathy
Source: Front Immunol. 2026 May 11;17:1715735. doi: 10.3389/fimmu.2026.1715735 (PMC13199282; doi:10.3389/fimmu.2026.1715735)
Supplement: Supplementary file 1 [file Table1.docx]

**Supplementary Materials**

**Supplementary Methods**

**In situ hybridization of miRNAs in renal tissue**

Renal tissue fixed in 4% paraformaldehyde was embedded in paraffin, sectioned, and then deparaffinized in xylene and hydrated in graded dilutions of ethanol. For antigen recovery, paraffin sections were placed in 1× citric acid buffer (pH 6.0) and heated in a microwave at high power for 10 min, left at room temperature for 10 min, then heated at medium power for 10 min, before again being cooled to room temperature. Next, 3% H_2_O_2_ was added to the sections in a dropwise manner, and then they were incubated at room temperature in the dark for 15 min to block endogenous peroxidase activity. Pre-hybridization solution (1:100 dilution in salmon semen) was then added in a dropwise manner, and the sections were incubated at 37°C for 1 h. Subsequently, the sections were incubated with 2 µM hsa-miR-142-3p probe hybridization solution (added in a dropwise manner) overnight at 42°C, then washed at 37°C for 10 min in gradient citrate buffer solutions (4×, 2×, and 1×). Subsequently, digoxin-labeled peroxidase was added dropwise and the sections were incubated at 37°C for 1 h, then freshly prepared DAB substrate solution was added dropwise, and brownish-yellow staining indicated expression. Counterstaining was performed with hematoxylin for 3 minutes, 1% hydrochloric acid–alcohol was added for a few seconds, and then ammonia solution was added. The sections were then dehydrated in increasing concentrations of ethanol, mounted, and analyzed by light microscopy.

**Luciferase reporter assay**

Potential target genes of miR-142-3p were predicted using diana_microt (http://www.microrna.gr/microT), and INPP5F was identified as a gene of interest for further research. HK-2 cells (10^5^) were transfected with 3′-untranslated region (UTR) luciferase reporter constructs (3′UTR-NC, 3′UTR-INPP5F, or 3′UTR-TINPP5F-mutant), miRNA constructs (miRNA-NC or miR-142-3p-mimic), and a Renilla luciferase-expressing construct using Lipofectamine 2000 for 24 h. The subsequent luciferase activities were measured consecutively using a Dual-Luciferase Reporter Assay Kit (Beyotime Biotechnology, Naimen, China). These experiments were independently performed on three occasions.

**RT-PCR**

The sequences of siINPP5F-1, siINPP5F-2, siINPP5F-3, and the control small interfering RNAs (siRNA) were as follows:

(1) siINPP5F-1:

sense: 5’-CCAAUGUGUCUGCUCCUAATT-3’

antisense: 5’-UUAGGAGCAGACACAUUGGTT-3’

(2) siINPP5F-2:

sense: 5’-GCACAGAUAGUAGCGUUCATT-3’

antisense: 5’-UGAACGCUACUAUCUGUGCTT-3’

(3) siINPP5F-3:

sense: 5’-GAGAAUGUUCAGACACUAATT-3’

antisense: 5’-UUAGUGUCUGAACAUUCUCTT-3’

(4) siRNA control:

sense: 5’-UUCUCCGAACGUGUCACGUTT-3’

antisense: 5’-ACGUGACACGUUCGGAGAATT-3’.

**Western blotting**

The membranes were blocked with 5% bovine serum albumin for 1 h, then incubated with the following primary antibodies overnight: anti-INPP5F (Sc-514657, 1:1,000, Santa Cruz), anti-Fibronectin (26836T, 1:1,000, Cell Signaling Technology), anti-E-cadherin (3195T, 1:2,000, Cell Signaling Technology), anti-alpha-smooth muscle actin (α-SMA, 19245T, 1:1,000, Cell Signaling Technology), anti-Collagen I (Ab138492, 1:1,000, Abcam), anti-Collagen III (Ab184993, 1:1,000, Abcam), anti-PI3K (710400, 1:1,000, Invitrogen), anti-p-PI3K (Ab278545, 1:1,000, Abcam), anti-AKT (4691, 1:1,000, Cell Signaling Technology), anti-p-AKT (4060, 1:1,000, Cell Signaling Technology), or anti-GAPDH (AF7021, 1:5,000, Affinity). Goat anti-rabbit secondary antibody (bs-0295G-HRP, Bioss) and a ChemiScope6100 (Clinx Science Instruments) were used to identify specific bands. The relative expression of each protein was normalized to that of GAPDH.

**Supplementary Table 1****.** Baseline characteristics of chip cohort.

|  | Total cohort | Progression | Non-progression | P value |
| --- | --- | --- | --- | --- |
| Number of patients | 18 | 6 | 12 |  |
| Gender (M/F) | 9/9 | 3/3 | 6/6 | 1.000 |
| Age (year) | 27.50 (25.00-39.50) | 34.50±10.39 | 30.50±8.55 | 0.396 |
| Serum creatinine (µmol/L) | 98.03±33.74 | 90.47±40.43 | 101.81±31.14 | 0.518 |
| eGFR (ml/min.1.73m^2^) | 89.05±28.74 | 96.55±33.12 | 85.30±27.05 | 0.451 |
| Proteinuria (g/d) | 1.14 (0.38-1.97) | 2.48±1.27 | 0.80±0.75 | 0.003 |
| Follow-up duration (year) | 9.66 (9.48-9.69) | 9.59 (8.52-9.87) | 9.66 (9.51-9.68) | 0.639 |
| Oxford classification |  |  |  |  |
| M0/M1 | 11/7 | 4/2 | 7/5 | 1.000 |
| E0/E1 | 17/1 | 5/1 | 12/0 | 0.333 |
| S0/S1 | 5/13 | 1/5 | 4/8 | 0.615 |
| T0/T1/T2 | 9/3/6 | 1/1/4 | 8/2/2 | 0.131 |

**Supplementary Table 2.** Differential expression profiles of urinary sediment miRNAs between IgAN progression group and non-progression group.

| miRNAs | Fold change | P value |
| --- | --- | --- |
| hsa-mir-4304 | 1.56 | 0.0027 |
| hsa-miR-5580-3p | 1.81 | 0.0027 |
| hsa-miR-22-5p | 1.84 | 0.0151 |
| hsa-miR-3605-5p | 1.52 | 0.0204 |
| hsa-miR-4283 | 0.65 | 0.0316 |
| hsa-miR-29b-1-5p | 1.67 | 0.0323 |
| hsa-mir-6886 | 0.61 | 0.0359 |
| hsa-mir-6789 | 0.64 | 0.0371 |
| hsa-miR-3192-5p | 1.66 | 0.0373 |
| hsa-miR-29b-3p | 2.41 | 0.0452 |
| hsa-miR-196b-3p | 2.07 | 0.0456 |
| hsa-miR-3163 | 1.24 | 0.0002 |
| hsa-miR-302b-5p | 0.83 | 0.0008 |
| hsa-miR-4709-3p | 0.68 | 0.0038 |
| hsa-mir-4727 | 1.20 | 0.0088 |
| hsa-miR-548az-3p | 1.21 | 0.0094 |
| hsa-miR-4457 | 1.22 | 0.0108 |
| hsa-miR-190a-3p | 1.25 | 0.014 |
| hsa-mir-6892 | 1.31 | 0.0163 |
| hsa-mir-5680 | 1.22 | 0.0187 |
| hsa-miR-4714-5p | 1.20 | 0.0193 |
| hsa-mir-6855 | 1.32 | 0.0212 |
| hsa-mir-4444-1 | 0.82 | 0.0256 |
| hsa-mir-4444-2 | 0.82 | 0.0256 |
| hsa-mir-602 | 0.77 | 0.0275 |
| hsa-miR-4772-5p | 1.20 | 0.0276 |
| hsa-miR-4761-5p | 0.81 | 0.0281 |
| hsa-mir-452 | 1.36 | 0.0311 |
| hsa-miR-142-3p | 1.27 | 0.0323 |
| hsa-miR-217 | 1.23 | 0.0343 |
| hsa-mir-4717 | 0.68 | 0.0378 |
| hsa-miR-4756-3p | 1.27 | 0.0396 |
| hsa-miR-6802-3p | 1.23 | 0.0415 |
| hsa-mir-224 | 1.27 | 0.0441 |
| hsa-miR-4478 | 0.71 | 0.0454 |
| hsa-miR-142-5p | 1.30 | 0.0471 |

**Supplementary Table 3.** miRNAs with similar trends in urinary sediment and renal tissue microarray results between IgAN progression group and non-progression group.

|  | miRNA expression profile of urinary sediment (18) | | miRNA expression profile in renal tissue (13) | |
| --- | --- | --- | --- | --- |
|  | Fold change (progression/non-progression) | P value | Fold change (progression/non-progression) | P value |
| miR-142-3p | 1.27 | 0.0323 | 4.66 | 0.0116 |
| miR-142-5p | 1.30 | 0.0471 | 3.32 | 0.0301 |

**Supplementary Table 4.** Baseline characteristics of training cohort.

|  | Total cohort | Progression | Non-progression | P value |
| --- | --- | --- | --- | --- |
| Number of patients | 165 | 53 | 112 |  |
| Gender (M/F) | 80/85 | 21/32 | 59/53 | 0.117 |
| Age (year) | 33.00 (27.00-41.00) | 35.00 (26.00-42.00) | 33.00 (27.00-41.00) | 0.769 |
| MAP (mmHg) | 95.33 (89.50-103.33) | 99.58±13.84 | 95.19±8.91 | 0.038 |
| Hematuria (%) | 86.06 | 88.68 | 84.82 | 0.504 |
| Serum albumin (g/L) | 39.30 (35.70-41.60) | 35.90 (31.38-38.85) | 40.35 (37.70-42.55) | <0.001 |
| Serum creatinine (µmol/L) | 93.70 (72.95-126.85) | 144.10 (101.83-214.70) | 84.70 (65.05-106.03) | <0.001 |
| eGFR (ml/min.1.73m^2^) | 87.48 (57.64-113.09) | 53.47 (33.36-85.29) | 99.24 (78.27-117.50) | <0.001 |
| Proteinuria (g/d) | 1.22 (0.65-2.21) | 2.15 (1.57-3.07) | 0.95 (0.59-1.66) | <0.001 |
| Urinary NAG (U/L) | 23.25 (14.83-34.28) | 25.10 (16.70-35.23) | 22.00 (14.65-33.28) | 0.246 |
| Follow-up duration (year) | 5.93 (3.76-8.33) | 6.19 (3.84-8.24) | 5.90 (4.30-8.24) | 0.528 |
| Oxford classification |  |  |  |  |
| M0/M1 | 80/85 | 14/39 | 66/46 | <0.001 |
| E0/E1 | 148/17 | 45/8 | 103/9 | 0.164 |
| S0/S1 | 68/107 | 10/43 | 48/64 | 0.003 |
| T0/T1/T2 | 83/41/41 | 8/18/27 | 75/23/14 | <0.001 |
| C0/C1/C2 | 105/59/1 | 34/18/1 | 71/41/0 | 0.925 |
| ACEI/ARB usage rate (%) | 81.21 | 73.58 | 84.82 | 0.084 |
| Immunosuppressive agents usage rate (%) | 10.30 | 11.32 | 9.82 | 0.767 |

MAP, mean arterial pressure; NAG, β-N-Acetyl-D-glucosaminidase; ACEI, angiotensin-converting enzyme inhibitor; ARB, angiotensin receptor blocker.

**Supplementary Table 5****.** Features of the receiver operating characteristic curve of urinary sediment miR-142-3p for prediction of IgAN progression from training cohort.

|  | AUC | 95% CI | P value | Sensitivity (%) | Specificity (%) |
| --- | --- | --- | --- | --- | --- |
| miR-142-3p | 0.796 | 0.724-0.869 | <0.0001 | 75.5 | 70.5 |

AUC, the area under the ROC curve; CI, confidence interval.

**Supplementary Table 6.** Baseline characteristics of disease control group training cohort.

|  | Total cohort | Progression | Non-progression | P value |
| --- | --- | --- | --- | --- |
| Number of patients | 70 | 11 | 59 |  |
| Gender (M/F) | 31/39 | 6/5 | 25/34 | 0.520 |
| Age (year) | 37.24±14.67 | 34.00 (26.00-35.00) | 31.00 (26.25-42.75) | 0.534 |
| MAP (mmHg) | 96.20±11.60 | 98.33±12.63 | 95.80±11.47 | 0.510 |
| Hematuria (%) | 40.00 | 54.55 | 37.29 | 0.328 |
| Serum albumin (g/L) | 24.94±5.64 | 25.56±4.11 | 24.83±5.90 | 0.694 |
| Serum creatinine (µmol/L) | 72.75 (56.60-93.90) | 93.63±45.31 | 82.18±39.41 | 0.391 |
| eGFR (ml/min.1.73m^2^) | 111.11 (82.79-130.37) | 100.22±44.77 | 104.90±32.61 | 0.683 |
| Proteinuria (g/d) | 5.25 (3.51-6.94) | 5.00±1.82 | 5.87±2.80 | 0.326 |
| Urinary NAG (U/L) | 35.50 (19.53-73.08) | 12.80 (8.09-34.40) | 38.90 (21.55-85.30) | 0.171 |
| Follow-up duration (year) | 5.16 (3.46-7.29) | 5.94±2.79 | 5.26±2.40 | 0.407 |

MAP, mean arterial pressure; NAG, β-N-Acetyl-D-glucosaminidase.

**Supplementary Table 7.** Baseline characteristics of validation cohort.

|  | Total cohort | Progression | Non-progression | P value |
| --- | --- | --- | --- | --- |
| Number of patients | 146 | 10 | 136 |  |
| Gender (M/F) | 80/66 | 7/3 | 73/63 | 0.502 |
| Age (year) | 34.00 (29.00-42.00) | 30.50 (25.75-43.25) | 34.00 (30.00-41.75) | 0.416 |
| MAP (mmHg) | 96.33 (88.00-105.58) | 100.33±16.39 | 96.67±14.21 | 0.438 |
| Hematuria (%) | 79.45 | 50.00 | 81.62 | 0.047 |
| Serum albumin (g/L) | 39.35 (36.78-42.50) | 37.55 (34.80-41.63) | 39.40 (37.03-42.50) | 0.367 |
| Serum creatinine (µmol/L) | 100.35 (80.25-127.88) | 219.90 (126.43-283.78) | 97.95 (78.75-123.45) | <0.001 |
| eGFR (ml/min.1.73m^2^) | 79.66±27.86 | 36.32 (26.12-49.96) | 83.16 (61.30-100.40) | <0.001 |
| Proteinuria (g/d) | 1.05 (0.82-2.25) | 4.00 (0.66-5.70) | 1.04 (0.83-2.14) | 0.014 |
| Urinary NAG (U/L) | 20.55 (13.45-27.88) | 15.10 (10.70-25.90) | 20.70 (13.50-28.40) | 0.420 |
| Follow-up duration (year) | 2.48 (1.75-3.64) | 2.04 (1.31-2.66) | 2.50 (1.76-3.74) | 0.483 |
| Oxford classification |  |  |  |  |
| M0/M1 | 77/69 | 5/5 | 72/64 | 1.000 |
| E0/E1 | 121/25 | 7/3 | 114/22 | 0.493 |
| S0/S1 | 34/112 | 2/8 | 32/104 | 1.000 |
| T0/T1/T2 | 89/50/7 | 2/4/4 | 87/46/3 | 0.016 |
| C0/C1/C2 | 94/48/4 | 5/4/1 | 89/44/3 | 0.521 |
| ACEI/ARB usage rate (%) | 78.77 | 50.00 | 80.88 | 0.057 |
| Immunosuppressive agents usage rate (%) | 0.68 | 0 | 0.74 | 1.000 |

MAP, mean arterial pressure; NAG, β-N-Acetyl-D-glucosaminidase; ACEI, angiotensin-converting enzyme inhibitor; ARB, angiotensin receptor blocker.

**Supplementary Table 8.** Features of the receiver operating characteristic curve of urinary sediment miR-142-3p for prediction of IgAN progression from validation cohort.

|  | AUC | 95% CI | P value | Sensitivity (%) | Specificity (%) |
| --- | --- | --- | --- | --- | --- |
| miR-142-3p | 0.779 | 0.632-0.925 | <0.001 | 50.0 | 97.1 |

AUC, the area under the ROC curve; CI, confidence interval.

**Supplementary Table 9.** Baseline characteristics of disease control group validation cohort.

|  | Total cohort | Progression | Non-progression | P value |
| --- | --- | --- | --- | --- |
| Number of patients | 36 | 5 | 31 |  |
| Gender (M/F) | 19/17 | 4/1 | 15/16 | 0.342 |
| Age (year) | 43.81±15.65 | 56.20±9.09 | 41.81±15.65 | 0.018 |
| MAP (mmHg) | 94.18±12.39 | 102.20±10.29 | 92.88±12.35 | 0.120 |
| Hematuria (%) | 38.89 | 40.00 | 38.71 | 1.000 |
| Serum albumin (g/L) | 29.05±6.50 | 29.34±5.00 | 29.00±6.78 | 0.917 |
| Serum creatinine (µmol/L) | 71.75 (59.01-106.93) | 62.96 (51.51-95.78) | 72.70 (62.35-119.60) | 0.423 |
| eGFR (ml/min.1.73m^2^) | 99.42±36.04 | 110.82±22.61 | 97.58±37.71 | 0.454 |
| Proteinuria (g/d) | 3.47±2.37 | 4.96±3.45 | 3.23±2.13 | 0.131 |
| Urinary NAG (U/L) | 30.00 (20.13-51.63) | 35.40 (29.48-80.85) | 28.25 (19.13-51.63) | 0.419 |
| Follow-up duration (year) | 1.73 (1.19-1.91) | 1.57±0.49 | 1.68±0.61 | 0.688 |

MAP, mean arterial pressure; NAG, β-N-Acetyl-D-glucosaminidase.

**Supplementary Table 10.** Baseline characteristics of IgAN renal tissue mass spectrometry cohort.

|  | Total cohort | Progression | Non-progression | P value |
| --- | --- | --- | --- | --- |
| Number of patients | 59 | 6 | 48 |  |
| Gender (M/F) | 26/28 | 1/5 | 25/23 | 0.194 |
| Age (year) | 30.00 (26.00-35.00) | 28.00 (26.25-31.25) | 30.00 (26.00-40.00) | 0.710 |
| MAP (mmHg) | 95.03±12.08 | 89.22±18.64 | 95.28±11.59 | 0.266 |
| Hematuria (%) | 93.22 | 100.00 | 91.67 | 1.000 |
| Serum albumin (g/L) | 39.78±4.19 | 37.80±5.12 | 40.29±4.04 | 0.172 |
| Serum creatinine (µmol/L) | 78.80 (66.90-96.50) | 64.60 (55.80-84.80) | 74.00 (66.90-91.80) | 0.680 |
| eGFR (ml/min.1.73m^2^) | 103.55±28.09 | 102.79±25.65 | 105.69±26.53 | 0.801 |
| Proteinuria (g/d) | 0.96 (0.60-1.67) | 0.98 (0.77-3.23) | 0.96 (0.59-1.57) | 0.349 |
| Oxford classification |  |  |  |  |
| M0/M1 | 35/24 | 3/3 | 29/19 | 0.658 |
| E0/E1 | 46/13 | 4/2 | 37/11 | 0.955 |
| S0/S1 | 18/41 | 1/5 | 14/34 | 1.000 |
| T0/T1/T2 | 38/17/4 | 5/0/1 | 31/16/1 | 0.651 |
| Follow-up duration (year) | 4.99±1.85 | 5.05±2.17 | 4.98±1.83 | 0.938 |

MAP, mean arterial pressure.


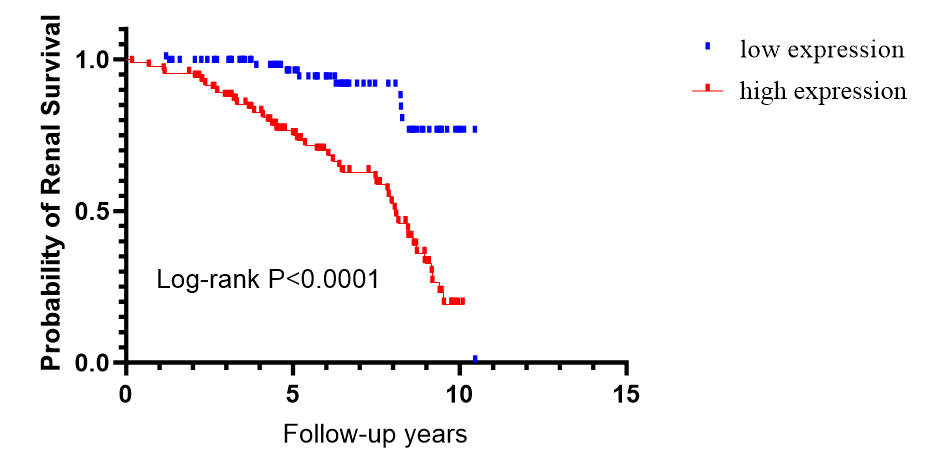


**Supplementary Figure 1.** Renal survival analysis of IgAN patients stratified by urinary miR-142-3p expression levels.

**
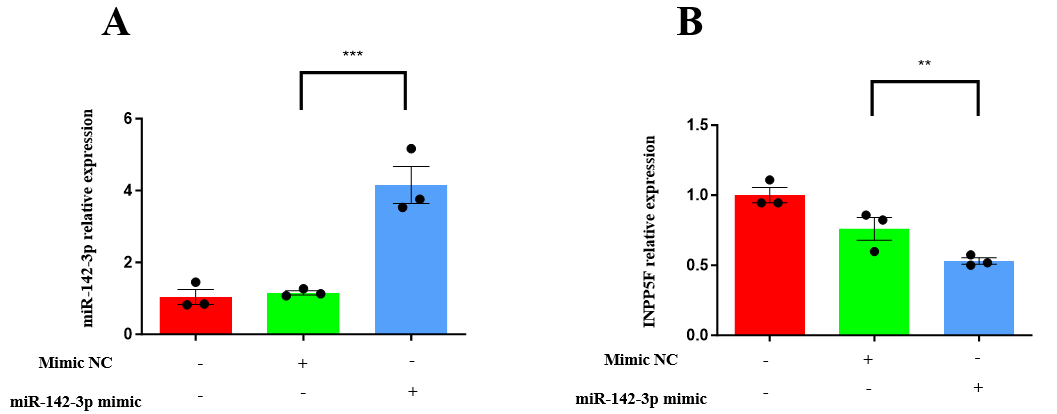
**

**Supplementary Figure 2.** RT-PCR analysis of miR-142-3p and INPP5F mRNA expression in HK-2 cells after transfection with miR-142-3p mimic.

**(A)** miR-142-3p expression status; **(B)** mRNA expression of INPP5F; HK-2+NC (HK-2 cells transfected with negative control mimic): HK-2+control mimic group; HK-2+miR-142-3p: HK-2+miR-142-3p mimic group. **, P<0.01, ***, P<0.001.


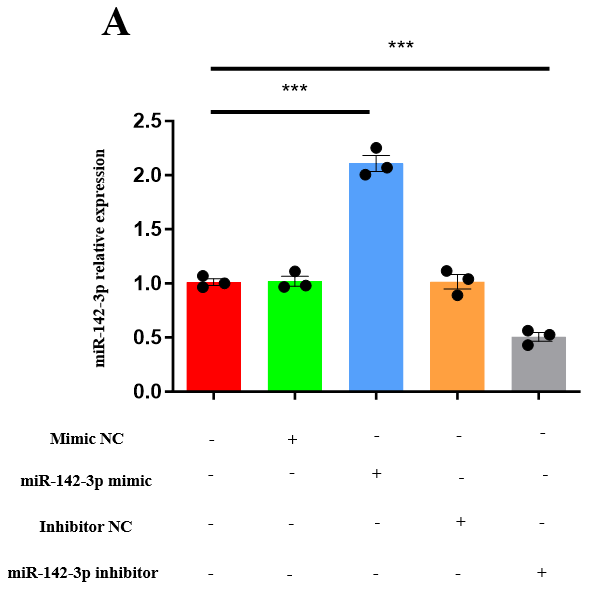


**Supplementary Figure 3** RT-PCR results of miR-142-3p mimic and miR-142-3p inhibitor intervention in HK-2 cells. ***, P<0.001.


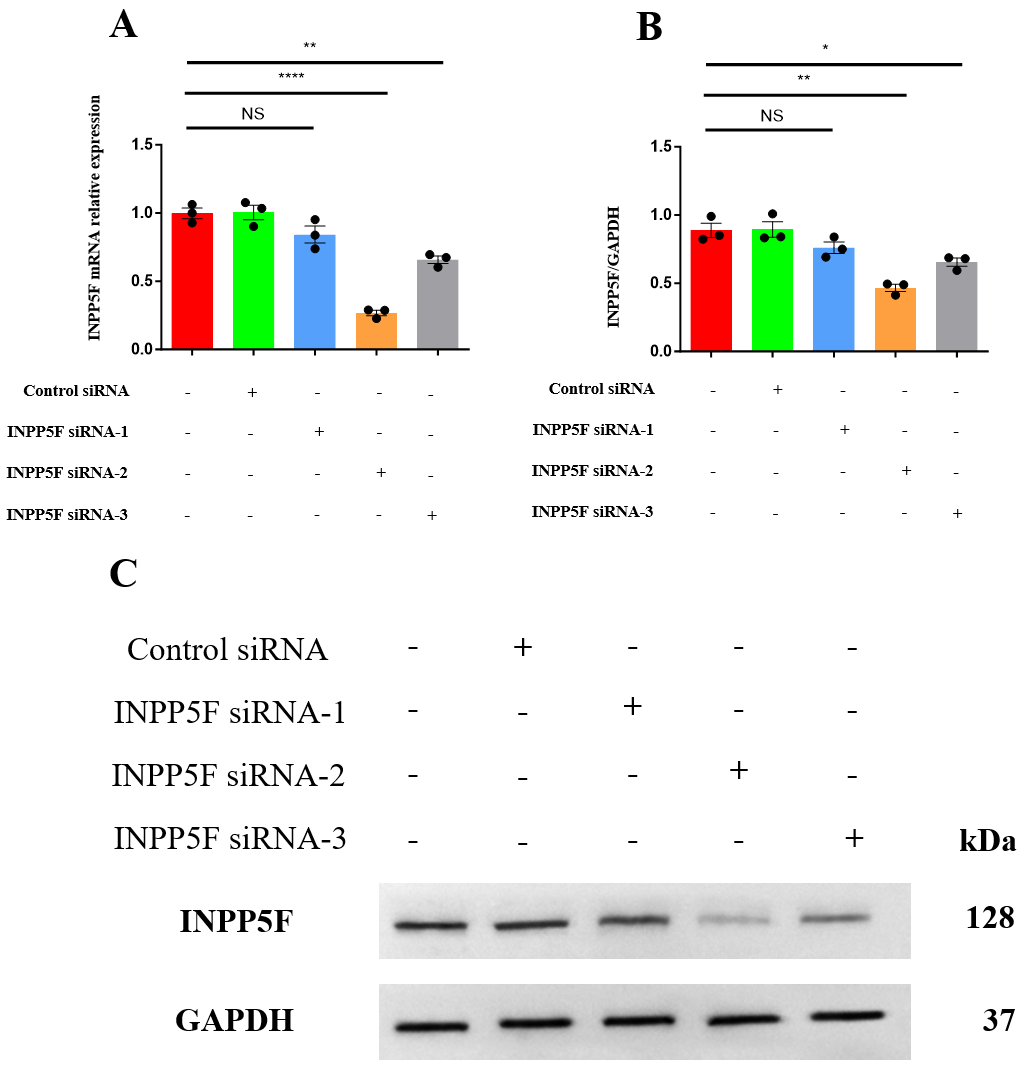


**Supplementary Figure 4** Differential expression of INPP5F after transfection with INPP5F siRNA.

1. mRNA expression of INPP5F; **(B)** Western blotting of INPP5F; **(C)** Western blotting of INPP5F subgroups. *, P<0.05, **, P<0.01, ****, P<0.0001, NS, no statistical difference.


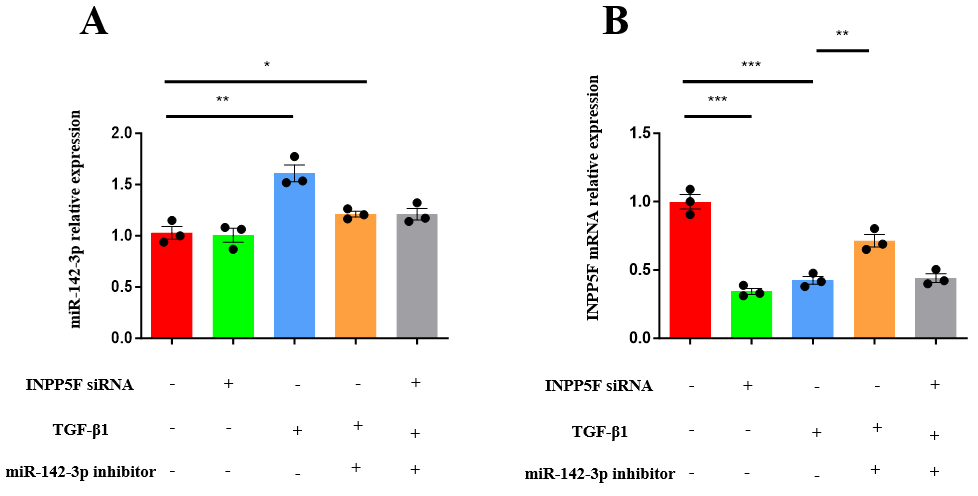


**Supplementary Figure 5** The RT-PCR results of miR-142-3p inhibitor intervention in HK-2 cells.

**(A)** miR-142-3p expression status; **(B)** mRNA expression of INPP5F. *, P<0.05, **, P<0.01, ***, P<0.001.
